# Parasitic Arthropods of Soricinae Shrews in North-Eastern Poland

**DOI:** 10.3390/ani13182960

**Published:** 2023-09-19

**Authors:** Grzegorz Karbowiak, Michal Stanko, Katerina Smahol, Joanna Werszko, Leszek Rychlik

**Affiliations:** 1 W. Stefański Institute of Parasitology, Polish Academy of Sciences, 00-818 Warsaw, Poland; horizont1254@gmail.com (K.S.); joanna.werszko@gmail.com (J.W.); 2 Faculty of Medical and Health Sciences, University of Social and Medical Sciences in Warsaw, 04-367 Warsaw, Poland; 3 Institute of Parasitology, Slovak Academy of Sciences, 040 01 Kosice, Slovakia; stankom@saske.sk; 4 Museum and Institute of Zoology, Polish Academy of Sciences, 00-679 Warsaw, Poland; 5 Department of General Biology and Parasitology, Medical University of Warsaw, 02-004 Warsaw, Poland; 6 Department of Systematic Zoology, Institute of Environmental Biology, Faculty of Biology, Adam Mickiewicz University, 61-614 Poznań, Poland; rychliklesz@gmail.com

**Keywords:** Soricidae, ticks, fleas, gamasid mites, parasite assemblage

## Abstract

**Simple Summary:**

Studies on the ectoparasites of insectivores are quite rare compared with other groups of mammals. The study of the ectoparasite fauna of the insectivores: *Sorex araneus*, *Sorex minutus*, *Neomys fodiens*, and *Neomys anomalus*—was carried out in three locations in north-eastern Poland: Białowieża National Park, Kosewo Górne in the Masurian Lake District, and one location in vicinity of Warsaw. Three species of ixodid ticks, eleven species of fleas and four species of mites from the order Mesostigmata were noted, in comparison to the total number of ectoparasite species on Soricinae, recorded in Central Europe, amounts to eighty-one species: six tick species, twenty-three gamasid mites, nine trombiculid mites, nine Myobiidae and Sarcoptiformes, twenty-nine flea species, and five sucking lice. The most numerous ectoparasites recorded during the study are ticks *Ixodes ricinus* (larvae), *Dermacentor reticulatus* (nymphs), fleas *Palaeopsylla soricis*, *Megabothris walkeri*, and *Hystrichopsylla orientalis*. These species show the highest prevalence and show the highest dominance index. The parasitofauna of *S. araneus* is much richer in species than other insectivorous species. The structure and dominance of parasite assemblages differ between locations.

**Abstract:**

The study of the ectoparasite fauna of the insectivores—*Sorex araneus*, *Sorex minutus*, *Neomys fodiens*, and *Neomys anomalus* (subfamily Soricinae)—was carried out in three locations in Poland: Białowieża National Park, Kosewo Górne in the Masurian Lake District, and in vicinity of Warsaw. Three species of Ixodidae ticks, eleven species of fleas, and four species of mites from the order Mesostigmata were noted. The most numerous ectoparasites are ticks *Ixodes ricinus* (larvae), *Dermacentor reticulatus* (nymphs), and fleas *Palaeopsylla soricis*, *Megabothris walkeri*, and *Hystrichopsylla orientalis*. These species show the highest prevalence and show the highest dominance index. The parasitofauna of *S. araneus* is much richer in species than other shrew species. The structure and dominance of parasite assemblages differ between locations.

## 1. Introduction

Soricinae shrews are a common and important component of the small mammal’s fauna in Central Europe. They play a role as small predators in the environment, and in the epidemiology of zoonotic diseases as zoonotic reservoir and/or amplifer of arthropod-borne pathogens, as *Babesia microti* [1,2,3] or some strains of *Anaplasma phagocytophilum* [4,5,6]. However, they are not abundant, thus their role as the source of infection for ticks is limited.

The parasitofauna of shrews is not very well known. There are available few data on the parasite species composition of particular species of shrews, mainly due to the fact that shrews are often captured during studies on parasites of small rodents. However, there are not many analyses on the structure of insectivorous parasite assemblages. The main reason is the high mortality of shrews in live-traps set for rodents, which results in blood-sucking arthropods leaving the surface of their bodies. In addition, many ectoparasites quickly leave dead animals, which further complicates parasitological studies. Trapping live insectivores is difficult and time-consuming [7,8,9,10], which makes their parasitological study difficult and not as popular as rodents.

## 2. Materials and Methods

In the article is summarised and analysed data on the blood-sucking arthropods collected from common shrews *Sorex araneus* Linnaeus, 1758, and Eurasian pygmy shrew *S. minutus* Linnaeus, 1766, accidentally caught during rodent parasite studies and water shrews—Iberian water shrew *Neomys anomalus* Cabrera, 1907 and Eurasian water shrew *N. fodiens* (Pennant, 1771), intentionally caught for behavioural research. The research was conducted in various regions of eastern and central Poland.

Long-term studies of the fauna of external arthropods associated with small mammals were carried out in the Białowieża Primeval Forest in 2005 (August), 2006 (July), 2007 (July), and 2008 (July), along the southern border of the National Park (52°42′29″ N, 23°52′42″ E) and in Kosewo Górne (53°49′12″ N 21°26′36″ E), commune of Mikołajki, Masurian Lake District in 2004 (August) and 2009 (July), south-eastern vicinity of Warsaw in 2011 (July) (52° 9′12.21″ N, 21°10′42.68″ E). The study in Masurian Lake District and Warsaw vicinity were conducted in areas including pinewood community *Leucobryo-Pinetum* habitat. The study in Białowieża were conducted in areas including the three following habitats: tussock-sedge swamp (*Caricetum appropinquatae* from alliance *Magnocaricion*), streamside alder-ash forest (*Circaeo-Alnetum* from alliance *Alno-Padion*) and wet grassland (with *Phalaris arundinacea* and *Bromus inermis*), reeds (*Phragmites communis*) and bushes (of *Salix* species), as well and the ecotone between them [11].

Small mammals were captured by using live in box-traps baited with oat seeds and minced beef. Live traps were set randomly overnight from transects and checked each morning and evening (Kosewo Górne and vicinity of Warsaw) or set in the evening and checked every three hours during the night (Białowieża). Arthropods were brushed from the fur of the mammals and harvested from the trap material and then preserved in 70% ethanol. In the case of live trapped shrews, after the parasites were examined and harvested, the mammals were released.

Additionally, *N. fodiens* and *N. anomalus* were trapped using cone traps, checked every four hours. As above, the arthropods were brushed from the fur of the mammals and harvested from the trap material and then preserved. Small mites and tick larvae were mounted on slides in Berlese liquid; adult ticks, lice, and fleas were identified immediately under a stereoscope and microscope or, if necessary, embedded in a Canadian balsam. The arthropods were identified to the respective species using the keys developed by Skuratowicz [12], Bregetova [13], Siuda [14], Mašán and Fenďa [15], and Wegner [16].

The structure of parasite groups was characterised by the indices of prevalence (P%) and intensity of infestation (I), the factors commonly used in the description of parasitocenoses. Prevalence, in some works known also as frequency indices, is expressed as a percentage ratio of the numbers of individuals of host species infected with a particular parasite species (or taxonomic group) to the number of hosts examined (number of hosts infected with a parasite species divided by the number of examined hosts) [17]. The calculation is made according to the formula *P* = (*N_p_*/*n*) × 100%, where *N_p_* = the number of hosts infected, *n* = the total number of hosts. I is the total number of parasites of a particular species found in a sample divided by the number of hosts infested with that parasite, according to the formula *I* = *Par/Np*, where *Par* = the number of parasites detected; *N_p_* = the number of infected hosts.

However, because there is often no correlation between the intensity of infestation and the prevalence of parasites, the index of dominance (D) is additionally used. D is calculated according to the formula *D_i_* = *n_i_* × 100/*N* [%], where: n_i_ = total number of parasites of a particular species, N—total number of all parasites. The index D can prove the dominant parasite species, following the subdominant and accessory species in the grouping [18,19], independent of the number of hosts and parasite population size. The structure of domination is classified according to Czachorowski [19], Kisielewska [20] and Margolis et al. [21]: Eu—eudominant (above 10%); Do—dominant (5.1–10.0%), Su—subdominant (2.1—5.0%), Re—rare (1.1–2.0%), and Ac—accidental (below 1.0%).

Following, the structural and quantitative characteristics of the parasitic arthropods communities were assessed, and the data were analysed using the abundance (A) indices according to Margolis et al. [21], Bush et al. [18], and Baláž and Zigová [22], calculated according to the formula A = Par/n, where Par = the number of parasites detected, and n = the number of hosts examined.

The analysis and comparison of the obtained results was limited to the data available for the countries of Central Europe, due to the potential differences in the geographic distribution of the parasite fauna [23,24].

## 3. Results

In total, 118 individuals of *Sorex araneus*, 17 *Sorex minutus*, 11 *Neomys fodiens*, and three *Neomys anomalus* were investigated. There were 19 species of parasitic arthropods identified—3 species of ixodid ticks: *Ixodes apronophorus* (Schulze, 1924), *Ixodes ricinus* (Linnaeus, 1758), *Dermacentor reticulatus* (Fabricius, 1794); 11 species of fleas: *Palaeopsylla soricis* (Dale, 1878), *Palaeopsylla similis* Dampf, 1910, *Ctenophthalmus agyrtes* (Heller, 1896), *Ctenophthalmus assimilis* (Taschenberg, 1880), *Ctenophthalmus bisoctodentatus* Kolenati, 1863, *Ctenophthalmus uncinatus* (Wagner, 1898), *Peromyscopsylla bidentata* (Kolenati, 1860), *Megabothris turbidus* (Rothschild, 1909), *Megabothris walkeri* (Rothschild, 1902), *Hystrichopsylla orientalis* Smit, 1956, *Doratopsylla dasycnema* (Rothschild, 1897); five species of mesostigmatid mites—*Eulaelaps stabularis* (Koch, 1836), *Laelaps hilaris* Koch 1836, *Haemogamasus hirsutosimilis* Willmann, 1952, *Haemogamasus nidi* Michael, 1892, and *Androlaelaps fahrenholzi* (Berlese, 1911).

The richest in species is the ectoparasite fauna of *S. araneus*. It includes all of the species listed above, except *I. apronophorus* (Table 1).

The ectoparasite fauna of *S. minutus* includes ticks *I. apronophorus*, *D. reticulatus*, fleas *P. soricis*, *P. bidentata*, *C. agyrtes*, *M. turbidus*, *M. walkeri*, and *H. orientalis* (Table 2).

On *N. fodiens* there were found ticks *I. apronophorus* and fleas *P. soricis*, on *N. anomalus*—fleas *P. soricis* and *M. walkeri* (Table 3). Ticks were present as larvae and nymphs.

The eudominant parasite of *S. araneus* in almost all localities of study was flea *P. soricis*; in the majority of the studied places, as eudominant a larvae and nymphs of *D. reticulatus* and *I. ricinus* ticks, and fleas *M. turbidus* and *M. walkeri* were found. Dominants in majority of studied places were three flea species—*P. similis*, *C. agyrtes*, and *H. orientalis*. Subdominats were fleas—*C. agyrtes*, *C. assimilis,* and mite *H. nidi*. Rare and accidental species were fleas *C. unicinatus*, *D. dasycnema*, mites *L. hilaris*, and *E. stabularis*. The structure of dominance coincides with the intensity of infestation and abundance.

Only in Kosewo Górne and Warsaw, in a forest environment, were mites from the order Mesostigmata found. The subdominant among them was *H.nidi*.

The dominance structure of parasitofauna of *S. minutus* is possible for material collected in Białowieża 2007; the material collected in 2005 is too poor to draw conclusions about the structure of the population. The eudominants are *D.reticulatus* nymphs, *M. walkeri*, and *H. orientalis* fleas. Dominants are *I. apronophorus*, *M. turbidus* and *P. soricis*. As in ectoparasites of *S. araneus*, the indices P, I, and A coincide. Lower categories cannot be distinguished on the basis of the obtained material (Table 2).

Parasitofauna of *N. fodiens* and *N. anomalus* is relatively poor, the numerous parasites are flea *P. soricis*, present on both water shrew species. The next parasite is *I. apronophorus*—larvae—on *N. fodiens* and *M. walkeri* on *N. anomalus* (Table 3).

The highest intensity of infestation shows the tick *D. reticulatus* nymphs, fleas *P. soricis*, *M. turbidus*, *M. walkeri*, and *C. agyrtes*, in Masurian District *I. ricinus* larvae and nymphs too.

The prevalence of ectoparasites was basically similar in all locations and catching periods. However, there was a noticeable difference in the tick’s infestation. The prevalence of the total of tick infestation was higher in August than in July. Moreover, there are different proportions between *I. ricinus* and *D. reticulatus*—*I. ricinus* infestation is higher in July, *D. reticulatus* infestation is higher in August. *I. ricinus* is present more often as larvae, *D. reticulatus* as nymphs. The records in July 2001 in Warsaw are a special case. The infestation I of *D. reticulatus* is 2.0 and 5.0 (larvae and nymphs, respectively) and the *I. ricinus* tick is absent on the area. Infestations with ectoparasites in particular years and localities are presented in Table 1.

The structure of parasitofauna of *S. araenus* is different in particular localities. The richest was in Białowieża in 2005, the number of parasite species affected *S. araneus* was 13. In 2007 there were seven species of parasitic arthropods noted, and in 2008 five species. In Kosewo Górne there were nine species and eight species noted in years 2004 and 2009, respectively. In Warsaw 2005 there were four species noted. The parasites noted in all studies were *D. reticulatus* nymphs and flea *P. soricis*, parasites noted in at least four of six studies were *I. ricinus* larvae and nymphs, fleas *M. turbidus*, *M. walkeri,* and *H. orientalis.*

*Sorex minutus*, *N. fodiens,* and *N. anomalus* are much less numerous, and therefore their participation in the study is smaller. Noteworthy, results were obtained in Białowieża in 2005 (August) and 2007 (July). Dominant parasites of *S. minutus* were fleas *P. soricis* and *H. orientalis* in both visits and in single studies, the dominants were *D. reticulatus* nymphs and *M. walkeri* fleas. In Białowieża 2007 there were noted larvae of *I. apronophorus* ticks, not recorded for *S. araneus*.

In the case of *N. fodiens* and *N. anomalus*, each of these mammals have two species of ectoparasites as eudominants. On *N. fodiens* were *I. apronophorus* tick larvae and flea *P. soricis*, on *N. anomalus* fleas *P. soricis* and *M. walkeri*. Due to the low number of samples and poor composition of parasite assemblage, the dominance structure is impossible to estimate.

## 4. Discussion

In our research carried out in the Masurian District, Białowieża Primeval Forest, and Warsaw vicinity, we confirmed the presence of 19 species of parasitic arthropods associated with shrew of the Soricinae subfamily. It is quite a high number, in comparison to the number of parasites noted in the whole Central Europe—81 [12,14,15,16,25,26,27,28]. We compare the four most common Insectivores species; *Sorex alpinus* and *Crocidura* spp. are not present or are very rare in north-eastern Poland, and therefore are omitted.

The structure of their taxonomic and ecological association (three tick species, eleven flea species and five Mesostigmatid mites) and proportions are typical of small insectivores in Central Europe and like earlier data published elsewhere (Table S1 in Supplementary Materials). Geographically, 13 species of ectoparasites occurring on *S. araneus, S. minutus, N. fodiens,* and *N. anomalus* are typical of the Palaearctic zone. These are ticks: *I. ricinus, I. apronophorus,* and *D. reticulatus*; mites: *L. hilaris* and *H. hirsutosimilis;* and fleas: *C. agyrtes, C. assimilis, C. uncinatus, M. turbidus, M. walkeri, P. soricis, P. bidentata,* and *D. dasycnema*. One species—*H. nidi* mite—occurs throughout the Holarctic zone, two species of fleas—*C. bisoctodentatus* and *Palaeopsylla similis*—occur only in Europe, flea *H. orientalis*—occurs in Central and Eastern Europe and Asia. Two species of mites—*E. stabularis* and *A. fahrenholzi*—has the cosmopolitan area of occurrence [12,14,29].

The mixed structure of arthropod communities associated with Soricidae populations in north-eastern Poland is characteristic of this biogeographic region, on the border of boreal and temperate forest zones. The Białowieża Primeval Forest is one of the last primeval forest complexes in Western and Central Europe and is particularly interesting place for biological research due to its geographical location on the border of boreal and temperate forest zones. This fact causes the mixing of fauna and flora of both zones [30]. The common shrew parasite fauna in Białowieża was the richest in species—it included 2 species of ticks and 11 species of fleas. These were parasites noted in studies also by other authors from Central Europe [15,22,23,30,31,32,33]. It is evident, that *I. ricinus* tick on *S. araneus* from Warsaw vicinity were absent; however, shrews were infested with *D. reticulatus*. It is the eastern components of parasitofauna (Table 1). Until the end of the 20th century, this tick species was present in Central Europe only east of the line of the Vistula and San rivers in Poland and Latorica river in Ukraine and Slovakia. Apart from Poland these ticks were found on *S. araneus* and *S. minutus* in Slovakia only, in the beginning of XXI century, since it started the expansion to the new areas [31,32].

The dominant group of ectoparasites—*I. ricinus* larvae and nymphs, *D. reticulatus* larvae and nymphs, *C. agyrtes*, *M. walkeri,* and *H. orientalis* fleas—includes typical dominants for small mammals in this region of Europe [22,26,33]. The characteristic and dominant component of flea communities are *P. soricis*, and *M. walkeri* fleas. *P. soricis* is a species strongly associated to insectivores, present also on other small mammals but in lower prevalence [33,34]. Moreover, the dominance of other flea species is typical for other records. Rosický and Černý [35] showed the dominance of *P. soricis*, followed by *C. assimilis, P. similis*, *D. dasycnema*, *C. agyrtes*, and *H. talpae*. The eudominant mite was *E. stabularis*, the subdominant was *H. nidi*. These mites are common and dominate in the parasite fauna of *S. araneus* and common on other Soricidae according to other authors [29,33,34,36].

Szabo [37], obtained a similar order to that obtained in our study, except for fleas *D. dasycnema*, a species quite rare in Poland. An interesting fact is the high prevalence of the flea *M. walkeri.* It is a species strongly associated to the root vole *Microtus oeconomus* (Pallas, 1776) [12,38,39,40] and is present according to the presence of that host. On other species of small mammals it occurs less frequently, and only in populations coexisting with *M. oeconomus*. In the study, the flea was present in Białowieża and Kosewo Górne, where the root vole occurs too, and does not occur in Warsaw vicinity, where *M. oeconomus* is absent. In Central Europe south of the Carpathian range, this species occurs locally on insectivorous mammals, only where *M. oeconomus* occurs. An example is the presence of *M. walkeri* flea on *S. araneus* and *N. fodiens* in the area of Kiz Balaton, where there is a relict population of *M. oeconomus mehelyi* [38,40]. Flea *D. dasycnema* is recorded by other authors as common on Soricidae [12,26,41,42], and on the study described it was found in Białowieża only. This is a relatively rare species in Poland although typical for *Sorex* shrews [43], and highly present in the other area of their occurrence [44]. Another species of note is *I*. *trainguliceps*. It is reported as a common species in Slovakia [45,46,47] but was not found during our research. This tick is associated with the highland regions, in Poland it is found in the south of the country, but in the central and northern regions it is absent or rare. However, it is common on small mammals in Northern Europe [14,23].

No species typical for other zones were found. Boreo-Alpine species found on insectivores in Austria have not been reported. These are two boreo-alpine flea species—*Amphipsylla rossica* and *A. sibirica* present in Central Europe only in insectivores in mountain areas in Austria and the Czech Republic. It concerns also *I. trianguliceps* tick [34,48].

*D. marginatus* and *Haemaphysalis concinna* ticks are common south of the Carpathian Mountains and recorded in insectivores [49,50,51] while to the north they are practically absent.

The high prevalence of *D. reticulatus* ticks on *Sorex araneus*, mainly nymphs, is in accordance with the developmental cycle of this tick [52]. The study was confirmed in July–August, the months of the immature stages of this tick activity. During a study conducted by Karbowiak [39] in Masurian District in July, the infestation of the bank voles *Myodes glareolus* (Schreber, 1780) with larvae and nymphs of *D. reticulatus* reached 43.0%, the infestation of the yellow-necked mice *Apodemus flavicolis* (Melchior, 1834), occurring in the same area, was 27.0%, the infestation of *M. oeconomus* reach 100%. Moreover, the infestation of other small mammals is the highest in July and in August [39,53]. This phenomenon is related to the short feeding period of the juvenile stages of *D. reticulatus*. Outside these months, immature stages of *D. reticulatus* no longer occur during the year [14,39,52,53].

The prevalence of infestation with *I. ricinus* on *S. araneus* in Białowieża is relatively low, and high in Kosewo Górne. The first possible explanations are environmental differences. *D. reticulatus* prefers open areas, so inevitably, their residents will be frequent. The shrew inhabits woodland, where these ticks are less numerous and show higher prevalence of *I. ricinus*.

Among ticks, *I. apronophorus* is recorded in Poland on *S. araneus* [54] but is a new species associated with *N. fodiens*, although both water shrew species are associated with wet habitats [11,55]. On *S. minutus*, this species was not recorded. The occurrence and distribution of *I. apronophorus* ticks are poorly known in Poland and throughout Europe. Currently, it is considered a rare species, but it is possible that further research will reveal its wider distribution and host range than described so far [53,54,56,57].

*H. orientalis* is a species associated with moles mainly; however, often affects small insectivores, especially inhabited in humid environments. It is noted from *S. araneus*, *N. fodiens,* and *N. anomalus* [12,26,58,59,60]. On *S. minutus* was noted so far from Poland only [59,60].

The parasite fauna of *S. minutus* and *N. fodiens* and especially *N. anomalus* is definitely poorer than that of *S. araneus*. This is evident both in the reported results and in the review of available data from Central Europe. The less diverse fauna of *Neomys* sp. compared to other small mammals was also pointed out by Haitlinger [60]. An explanation may be the semi-aquatic lifestyle of *Neomys fodiens* and *N. anomalus*. These mammals spend most of their lives submerged in water, which is a limiting factor for most parasitic arthropods sensitive to water. The presence of the *I. apronophorus* tick, associated with wetlands, may be evidence of this. This tick was found on various species of mammals, but always in swampy areas.

## 5. Conclusions

The total number of ectoparasite species on Soricinae, recorded in Central Europe, amounts to 81 species: 6 tick species, 23 gamasid mites, 6 trombiculid mites, 6 Myobiidae and Sarcoptiformes, 29 flea species, five sucking lice [15,22,23,25,26,29,30,33,36,61] (Table S1 in Supplementary Materials). A significant proportion of these reports are single finds. Out of total 69 ectoparasites of *S. araneus*, six, 38, 28 and five species of ticks, mites, fleas, and sucking lice species are noted, respectively. Of these, 2, 18, 4, and 1 species were found on this only once, respectively, in total there are 25 single records; 10 species (one tick, five mite, three flea species) were recorded once only on *S. araneus*. Similarly for other insectivores species. In *S. minutus*, the total number of known ectoparasite species is 34 (3, 28, 19, 5 ticks, mites, fleas, and lice, respectively), 10 recorded once (1, 6, 2, 1 tick, mites, fleas, and lice, respectively). In *N. fodiens*, the total number of known ectoparasite species is 53 (3, 27, 19, 4 ticks, mites, fleas, and lice, respectively), 21 recorded once (1, 11, 8, 1 tick, mites, fleas, and lice, respectively; among these, 1 flea and 1 mite, and only on *N. fodiens*. In *N. anomalus*, the total number of known ectoparasite species is 34 (2, 20, 10, and 2 ticks, mites, fleas, and lice, respectively), 11 recorded once: 8 mite and three flea species. Thus, the number of arthropod ectoparasites found at least twice decreases to 53, 23, 33, and 23 in *S. araneus*, *S. minutus*, *N. fodiens,* and *N. anomalus*, respectively.

In our study, 197 species of parasitic arthropods were found. The most numerous ectoparasites of Soricinae are ticks *Ixodes ricinus*, as larvae, and *Dermacentor reticulatus*, as nymphs usually, fleas *Palaeopsylla soricis*, *Megabothris walkeri*, and *Hystrichopsylla orientalis* are commonly representative. These species show the highest prevalence and show the highest dominance index. The parasitofauna of *S. araneus* is much richer in species than other insectivorous species. The structure and dominance of parasite assemblages differ between locations.

All these species were recorded by other Authors in insectivores in other Central European countries. The differences concerned species more common south of the Carpathian range—such as the flea *D. dasycnema*, or trophically related to another host, such as the flea *M. walkeri*, whose main host is the root vole *M. oeconomus*.

The data cited certainly do not exhaust the list of blood-sucking arthropods related to insectivores; moreover, they are limited to the summer season only. Nevertheless, they show the most common species and may become useful in further studies.

## Figures and Tables

**Table 1:** Parasitic arthropods on *Sorex araneus*.

		Irc L	Irc N	Drt L	Drt N	Psr	Psm	Cag	Cas	Cbs	Cun	Pbd	Met	Mwl	Hor	Dds	Lah	Hhl	Han	Est	Anf
Białowieża August 2005																					
N = 40	ni nc	1 2		1 1	1 3	16 26	1 1	7 9	1 2	2 2	1 1	4 4	5 5	3 3	4 5	1 1					
Prevalence P %		2.5		2.5	2.5	40.0	2.5	17.5	2.5	5.0	2.5	10.0	12.5	7.5	10.0	2.5					
Intensity I		2.0		1.0	3.0	1.6	1.0	3.0	2.0	1.0	1.0	1.0	1.0	1.0	1.2	1.0					
dominance D		2.9		1.5	4.5	38.8	1.5	13.4	3.0	3.0	1.5	6.0	7.5	4.5	7.5	1.5					
abundance A		0.1		0.0	0.1	0.6	0.0	0.2	0.0	0.0	0.0	0.1	0.1	0.1	0.1	0.0					
Białowieża July 2007																					
N = 24	ni nc	2 2			4 8	2 6		1 1					4 6	1 2	1 1						
prevalence P %		9.1			18.1	9.1		4.6					18.2	4.6	4.6						
intensity I		1.0			2.0	3.0		1.0					1.5	2.0	1.0						
dominance D		7.7			30.08	23.1		3.8					23.1	7.7	3.8						
abundance A		0.1			0.4	0.3		0.1					0.3	0.1	0.1						
Białowieża July 2008																					
N = 10	ni nc			2 2	2 2	1 2				1 2	1 2		1 2								
prevalence P %				20.0	20.0	10.0				10.0	10.0		10.0								
intensity I				1.0	1.0	2.0				2.0	2.0		2.0								
dominance D				16.7	16.7	16.7				16.7	16.7		16.7								
abundance A				0.2	0.2	0.2				0.2	0.2		0.2								
Kosewo August 2004																					
N = 14	ni nc	3 3			2 2		2 2	4 12	3 4				2 3	3 3			2 3	2 2			
prevalence P %		21.4			14.3		14.3	28.6	21.4				14.3	21.4			14.3	14.3			
intensity I		1.0			1.0		1.0	7.0	1.0				1.0	0.7			1.0	1.0			
dominance D		15.8			10.5		10.5	63.2	21.0				15.8	15.8			15.8	10.5			
abundance A		0.2			0.1		0.1	0.9	0.3				0.2	0.2			0.2	0.1			
Kosewo July 2009																					
N = 11	ni nc	8 49	1 7		3 5	3 6			1 1						2 2				1 1	2 3	1 1
prevalence P %		72.7	9.1		27.3	27.3			9.1						18.2				9.1	18.2	9.1
intensity I		6.1	7.0		1.7	2.0			1.0						1.0				1.0	1.5	1.0
dominance D		65.3	9.3		6.7	8.0			1.3						2.7				0.3	04	1.3
abundance A		4.5	0.6		0.5	0.5			0.1						0.2				0.1	0.3	0.1
Warszawa July 2011																					
N = 19	ni nc			4 6	2 10	4 6		1 1											1 1		
prevalence P %				21.1	10.5	21.1		5.3											5.3		
intensity I				2.0	5.0	1.5		1.0											1.0		
dominance D				25.0	41.7	25.0		4.2											4.2		
abundance A				0.3	0.5	0.3		0.1											0.1		
In total																					
N = 118	ni nc	14 56	1 7	7 9	14 30	26.0 46	3 3	13 23	5 7	3 4	2 3	4 4	12 16	7 8	7 8	1 1	2 3	2 2	2 2	2 3	1 1
prevalence P %		11.9	0.9	5.9	11.9	22.0	9.5	11.0	4.2	2.5	1.7	3.4	10.2	6.0	2.6	0.9	1.7	1.7	1.7	1.7	0.9
intensity I		4.0	7.0	1.3	2.1	1.8	1.0	1.8	1.4	1.3	2.0	1.0	1.3	1.1	1.1	1.0	1.5	1.0	1.0	1.5	1.0
dominance D		6.3	0.4	3.1	6.3	11.7	1.4	5.8	2.2	1.4	0.9	1.8	5.4	3.1	3.2	0.4	0.9	0.9	0.9	0.9	0.4
abundance A		0.5	0.1	0.1	0.3	0.4	0.0	0.2	0.1	0.0	0.0	0.0	0.1	0.1	0.1	0.0	0.0	0.0	0.0	0.0	0.0

Abbreviations: N—total number of shrews investigated in a given year and locality; nc—number of parasite specimens collected; ni—number of shrews infested with a given ectoparasite; Anf—*A. fahrenholzi*; Cag—*C. agyrtes*; Cas—*C. assimilis*; Cbs—*C. bisoctodentatus*; Cun—*C. uncinatus*; Dds—*D. dasycnema*; Drt—*D. reticulatus*; Est—*E. stabularis*; Hhl—*H. hirsutosimilis*; Han—*H. nidi*; Hor—*H. orientalis*; Irc—*I. ricinus*; L—larvae; Lah—*L. hilaris*; Met—*M. turbidus*; Mwl—*M. walkeri*; N—nymphs; Pbd—*P. bidentata*; Psm—*P. similis*; Psr—*P. soricis*.

**Table 2:** Parasitic arthropods on *Sorex minutus*.

		Iap L	Drt N	Psr	Pbd	Cag	Met	Mwl	Hor
Białowieża August 2005									
N = 4	ni nc			1 1	1 1				1 1
Prevalence P %				25.0	25.0				25.0
Intensity I				1.0	1.0				1.0
dominance D				33.3	33.3				33.3
abundance A				0.25	0.25				0.25
Białowieża July 2007									
N = 13	ni nc	1 1	2 4	1 1		1 2	2 2	3 12	2 2
Prevalence P %		7.7	15.4	7.7		7.7	15.4	23.1	15.4
Intensity I		1.0	2.0	1.0		2.0	1.0	5.0	1.0
dominance D		4.2	16.7	4.2		8.3	8.3	50.0	8.3
abundance A		0.1	0.3	0.1		0.2	0.2	0.9	0.2
Total									
N = 17	ni nc	1 1	2 4	2 2	1 1	1 2	2 2	3 12	3 3
Prevalence P %		5.9	11.8	11.8	5.9	5.9	11.8	17.6	17.6
Intensity I		1.0	2.0	1.0	1.0	2.0	1.0	4.0	1.0
dominance D		3.7	7.4	7.4	3.7	3.7	7.4	11.1	11.1
abundance A		0.1	0.2	0.1	0.1	0.1	0.1	0.7	0.2

Abbreviations: N—total number of shrews investigated in a given year and locality; nc—number of parasite specimens collected; ni—number of shrews infested with a given ectoparasite; Cag—*C. agyrtes*; Drt—*D. reticulatus*; Hor—*H. orientalis*; Iap—*I. apronophorus*; L—larvae; Met—*M. turbidus*; Mwl—*M. walkeri*; N—nymphs; Pbd—*P. bidentata*; Psr—*P. soricis*.

**Table 3:** Parasitic arthropods on *Neomys fodiens and Neomys anomalus*. Białowieża, July 2007.

		Iap L	Psr	Mwal
*Neomys fodiens*				
N = 11	ni nc	1 3	5 20	
Prevalence P %		9.1	45.5	
Intensity I		3.0	4.0	
dominance D		4.3	21.7	
abundance A		0.3	1.8	
*Neomys anomalus*				
N = 3	ni nc		2 7	1 1
Prevalence P %			66.7	33.3
Intensity I			4.0	1.0
dominance D			25.0	12.5
abundance A			2.3	0.3

N—total number of shrews investigated in a given year and locality; nc—number of parasite specimens collected; ni—number of shrews infested with a given ectoparasite; Iap—*I. apronophorus*; L—larvae; Mwl—*M. walkeri*; Psr—*P. soricis.*

## Data Availability

The data presented in this study are available on request from the corresponding author.

## Supplementary Materials

The following supporting information can be downloaded at: https://www.mdpi.com/article/10.3390/ani13182960/s1, Table S1: List of *Sorex araneus*, *Sorex minutus*, *Neomys fodiens*, *Neomys anomalus* ectoparasites in Central Europe, according to various authors. References [62,63,64,65,66,67,68,69,70,71,72,73,74,75,76,77,78,79,80,81,82,83,84,85,86,87,88,89,90,91,92,93,94,95,96,97,98,99,100,101,102,103,104,105,106,107,108,109,110,111,112,113,114,115,116,117,118,119,120,121,122,123,124,125,126,127,128] are cited in the supplementary materials.
