# Supplementary material for: Parasitic Arthropods of Soricinae Shrews in North-Eastern Poland
Source: Animals (Basel). 2023 Sep 19;13(18):2960. doi: 10.3390/ani13182960 (PMC10525224; doi:10.3390/ani13182960)
Supplement: Supplementary file 1 [file animals-13-02960-s001.zip › animals-2521261-supplementary.pdf]

Article

Parasitic arthropods of Soricinae shrews in north-eastern Poland. Supplementary data.

Grzegorz Karbowiak[1,2*], Michal Stanko[3], Katerina Smahol[1,4], Joanna Werszko[1,5] and **Leszek Rychlik[6]**

[1] W. Stefański Institute of Parasitology, Polish Academy of Sciences, 00-818 Warsaw, Poland;grzgrz@twarda.pan.pl
2. University of Social and Medical Sciences in Warsaw, 04-367, Warsaw, Poland; g.karbowiak@usmbm.edu.pl
3. Institute of Parasitology, Slovak Academy of Sciences, 040 01, Kosice, Slovak Republic; stankom@saske.sk
4. Museum and Institute of Zoology, Polish Academy of Sciences, 00-679 Warsaw, Poland; horizont1254@gmail.com
5. Department of General Biology and Parasitology, Medical University of Warsaw, 02-004 Warsaw, Poland; joanna.werszko@gmail.com
6. Department of Systematic Zoology, Institute of Environmental Biology, Faculty of Biology, Adam Mickiewicz University, 61-614 Poznań, Poland; rychliklesz@gmail.com

* Correspondence: g.karbowiak@usmbm.edu.pl

Table S1. List of *Sorex araneus*, *Sorex minutus*, *Neomys fodiens*, *Neomys anomalus* ectoparasites in Central Europe, according to various authors. The borders of Central Europe according to Wojtanowicz [61] include:

Northern border- North Sea, Baltic;

Western border - Passage North Sea-Adriatic, Baltic – Black Sea; Rhine river;

Estern border - Dniepr and Dvina rivers;

Southern border – Black Sea in the South-East, Danube and Sava rivers, ridge of the Alps in the South-West.

Classification after Wegner [16], Skuratowicz [12], Krasnov [62], Krantz and Walter [63], and Guglielmone et al. [25].

	***Sorex araneus* Linnaeus, 1758**	***Sorex minutus* Linnaeus, 1766**	***Neomys fodiens* (Pennant, 1771)**	***Neomys anomalus* Cabrera, 1907.**
**Acari, Ixodidae**				
Dermacentor marginatus (Sulzer, 1776)	Slovakia [50]			
Dermacentor reticulatus (Fabricius, 1794)	Slovakia [32,50-51,64]	Slovakia [50,64]		
Haemaphysalis concinna Koch, 1844	Austria [65], Slovakia [49,51,66]	Slovakia [49]		
Ixodes apronophorus (Schulze, 1924)	Poland [14,54,], Slovakia [67]		Poland [14]	
Ixodes ricinus (Linnaeus, 1758)	Germany [5,68], Poland [14, 29,57,69-73], Czech [74], Slovakia [45,51,66,75-76]	Poland [14,29,57,69], Austria [65], Czech [74], Slovakia [66]	Poland [14,29,72-73], Czech [74], Slovakia [77]	Poland [59], Czech [74], Slovakia [74,77]
Ixodes trianguliceps Birula, 1895	Germany [5,68,78], Poland [29,70,72-73], Austria [79], Slovakia [45-47,80-82]	Poland [29,57,70,72-73], Austria [65,79], Slovakia [45-47,66,81-83]	Poland [29,70], Austria [65] Slovakia [45,66,77]	Poland [70], Slovakia [75-77]

**Acari, Ologamasidae**				
Cyrtolaelaps minor Willman, 1952:	Poland [72-73], Slovakia 77,84-87]			
Cyrtolaelaps mucronatus G. et R. Canestrini, 1881	Germany [88], Poland [29,70,72], Slovakia [46,77,84-86,90]	Poland [29,57,72]	Poland [29, 59, 72-73], Slovakia [77,83,85-86]	Poland [59], Slovakia [36], Hungary [91]
**Acari, Parasitidae**				
Androlaelaps fahrenholzi (Berlese, 1911)	Poland [57,70], Czech [92 (as *Haemolaelaps glasgowi*)], Slovakia [89-90]		Poland [59], Slovakia [77,85-86]	
Androlaelaps sardous Berlese, 1911	Slovakia [85-86]		Slovakia [85-86]	
Eulaelaps stabularis C.L.Koch, 1836	Germany [88], Poland [29,57-58,70,73], Slovakia [82,84-86,89]	Poland [29,58], Slovakia [84]	Poland [58,73], Slovakia [77,85-86,89]	Poland [58,70,73], Slovakia [36,86]
Haemogamasus ambulans (Thorell, 1872)	Slovakia [15]			
Haemogamasus bregetovae Mrciak, 1958	Poland [58]	Poland [58]	Poland [58]	Poland [58]
Haemogamasus hirsutosimilis Willmann, 1952	Poland [58,70], Czech [92], Slovakia [93]	Slovakia [93]	Poland [29-58], Slovakia [85-87,93]	Austria [94], Hungary [91]
Haemogamasus hirsutus Berlese, 1889	Poland [29,57-58,70-71,73], Czech [92], Slovakia [82,84,89]	Poland [58,70], Slovakia [85]	Poland [20, 32], Czech [92], Slovakia [36,77,85-86,93]	Poland [85,73], Hungary [91]
Haemogamasus horridus Michael, 1892	Germany [88 (as *Euhaemogamasus horridus*)], Poland [29,57-58,70,72-73], Austria [94], Slovakia [46,86]		Poland [58,72], Slovakia [85-86,77]	Poland [58,70], Slovakia [36], Hungary [91]

Haemogamasus nidi Michael, 1892	Poland [29,57,70-73], Austria [94], Slovakia [31,85-86,89,93]	Poland [70], Slovakia [93], Hungary [91]	Poland [72], Slovakia [36,77,85,87,89,93]	Poland [70,72-73], Slovakia [36,82,84-85], Hungary [91]
Hirstionyssus isabellinus (Oudemans, 1913) s. Evans et Till, 1966	Poland [29,57,70-71,73 (as *Echinonyssus isabellinus*)], Slovakia [30,46,76-77,82,84,95]	Slovakia [82,84]	Poland [72 (as *Echinonyssus isabellinus*)], Slovakia [93]	Poland [58]
Hirstionyssus soricis Turk, 1945	Poland [57,70-71, 73 (as *Echinonyssus soricis*)], Slovakia [82,84-85]	Poland [72,73 (as *Echinonyssus soricis*)], Slovakia [82,84]	Poland [29,73 (as *Echinonyssus soricis*)], Slovakia [77]	Poland [70,73 (as *Echinonyssus soricis*)], Slovakia [36,77], Hungary [91]
Hirstionyssus sunci Wang, 1962			Slovakia [77, 85, 93 (all as *H. apodemi*)]	
Holoparasitus coronarius Karg, 1971	Slovakia [84]			
Holoparasitus excipulciger (Berlese, 1905)	Slovakia [51]			
Hyperlaelaps microti (Ewing, 1953)	Poland [29,57,70-73], Czech [92 (as. *H. arvalis*)], Slovakia [85,93]	Poland [29,70], Slovakia [93]		Poland [70]
Laelaps agilis C.L.Koch, 1836	Poland [29,70,72-73], Austria [94], Slovakia [31,85-86,89,93,96]	Poland [29,73], Slovakia [31,86], Hungary [91]	Poland [29], Slovakia [36,85-86,77,93]	Poland [58], Slovakia [36,86,93], Hungary [91]
Laelaps clethrionomydis Lange, 1955	Slovakia [82,84-86,93,96]	Slovakia [82,84,93]	Slovakia [93]	Poland [58]
Laelaps hilaris Koch 1836	Poland [29,70-73], Austria [94], Czech [92], Slovakia [76-77,85-87,96]	Poland [29,70], Slovakia [82,84-87,93 42-45, 55]	Slovakia [77,85-86]	Poland [70], Slovakia [36]
Laelaps jettmari Vitzthum, 1930	Poland [57 (as *Laelaps pav-*			

	lovskyi)], Slovakia [89]			
Laelaps muris (Ljungh, 1799)	Austria [94], Slovakia [93]		Czech [92]	
Myonyssus ingricus Bregetova, 1956	Poland [57,70], Austria [94], Slovakia [82,84,93]	Poland [70]	Slovakia [31,36,93]	Poland [70,73], Slovakia [36,93], Hungary [91]
**Acari, Trombiculidae**				
Leptotrombidium europaeum (Daniel and Brehl, 1959)	Poland [70], Slovakia [97]			
Leptotrombidium silvaticum (Huscha, Schluger, 1967)			Poland [58]	
Trombicula (Neotrombicula) autumnalis (Shaw, 1790)	Germany [88], Poland [57,71,73], Slovakia [97]	Poland [70]	Poland [70-73]	Poland [73]
Neotrombicula inopinata (Oudemans, 1909)	Poland [72-73], Slovakia [82,97]	Poland [57], Slovakia [97]	Poland [73]	Poland [59], Slovakia [45,75]
Neotrombicula japonica (Tanaka, Kaiwa, Teramura and Kagaya, 1930)	Poland [57]			
Neotrombicula talmiensis (Schluger, 1955)	Poland [70], Slovakia [98]		Poland [73,93]	Poland [99]
Trombicula (Neotrombicula) zachvatkini (Schluger, 1948)	Poland [57,72 as *Hirsutiella zachvatkini*)]	Poland [57-70], Slovakia [45]	Poland [70]	
Neotrombicula vulgaris (Schluger, 1955)	Poland [99]		Poland [58]	
Radfordia lemnina (Koch, 1841)	Poland [57]			Poland [59]
**Acari, Myobiidae**				
Myobia musculi (Schranck, 1781)	Poland [57]			
Protomyiobia onoi Jameson and Dusbabek, 1971	Poland [71-73]			
Protomyiobia claparedei (Poppe,	Germany [88],			

1896)	Poland [71,73]			
**Acari, Sarcoptiformes**				
Amorphacarus elongatus (Poppe, 1896)	Germany [88, as *A. elongata*], Poland [70]			
Myocoptes japonensis (Radford, 1955)	Poland [27,57]		Poland [27,58]	
Myocoptes musculinus (Koch, 1844)	Poland [27]	Poland [27]	Poland [27]	
Trichoecius tenax (Michael, 1889)	Poland [27,57]			
Listrophorus brevipes Dubinina, 1968	Poland [27]	Poland [27]	Poland [27]	
Psorergates baueri Lukoschus, de Cock, Driessen, 1971			Austria [100]	
**Siphonaptera, Ceratophyllidae**				
Amalareus penicilliger (Grube, 1851)	Poland [72], Austria [48], Slovakia [76,82,101-102]			
Ceratophyllus hirundinis (Curtis, 1826)	Czech [103]			
Megabothris turbidus (Rothschild, 1909)	Poland [26,72-73,104 (as *Ceratophyllus turbidus*), 105], Czech [44, 106], Slovakia [22,33,38]		Slovakia [33,101,107-108]	Poland [105] Slovakia [38,101,107-108]
Megabothris walkeri (Rothschild, 1902)	Poland [26,71, 104 (as *Ceratophyllus walkeri*)]	Poland [104 (as *Ceratophyllus walkeri*), Hungary [33,40]	Hungary [33,40]	
Nosopsyllus fasciatus (Bosc, 1800)	Slovakia [38,109], Hungary [110]		Hungary [110]	Slovakia [38]
**Siphonaptera, Leptopsyllidae**				

Amphipsylla rossica Wagner, 1912.			Czech [44]	
Amphipsylla sibirica (Wagner 1898)	Austria [48]			
Leptopsylla segnis (Schönherr, 1811)	Poland [26], Czech [44 (as *Ctenopsyllus segnis*)]			
Peromyscopsylla bidentata (Kolenati, 1860)	Austria [48], Czech [44 (as *Ctenopsyllus bidentatus*), 106], Slovakia [32,82,108], Hungary [110]		Slovakia [101]	Slovakia [101-102,107-108], Hungary [110]
Peromyscopsylla fallax (Rothschild, 1909)	Slovakia [101,107], Hungary [111]			Hungary [111]
Peromyscopsylla silvatica (Meinert, 1896)	Poland [26,57], Czech [103]			
**Siphonaptera, Ctenophthalmidae**				
Ctenophthalmus agyrtes (Heller, 1896)	Germany [112], Poland [26,57,71,104-105,113], Austria [48,114], Czech [35,42,44,103,106], Slovakia [22,33,38,82,101-102] Hungary [33,40,110-111]	Poland [26,57,104-105], Czech [44], Slovakia [38,109,115]	Poland [26,104-105], Czech [42,44], Slovakia [82.101-102,107], Hungary [40,110]	Poland [105], Austria [48], Slovakia [38], Hungary [33,37,110]
Ctenophthalmus assimilis (Taschenberg, 1880)	Poland [26,57,71-72,104-105], Czech [35,42,44], Slovakia [22,33,38,101], Hungary [33,40,110]	Poland [104], Czech [44], Slovakia [38]	Poland [104-105], Czech [44], Slovakia [33,101,107], Hungary [33,40]	Slovakia [22,38], Hungary [110]
Ctenophthalmus bisoctodentatus Kolenati, 1863	Czech [44], Slovakia [101,107]		Poland [105]	

Ctenophthalmus congerer Rothschild, 1907	Poland [57], Austria [48]		Czech [44], Hungary [33]	
Ctenophthalmus obtusus Jordan et Rothschild 1912	Poland [113], Slovakia [116]		Poland [113]	
Ctenophthalmus solutus Jordan et Rothschild, 1920	Poland [105], Slovakia [38,107,101], Hungary [111,117]		Czech [44], Slovakia [101,107]	Slovakia [38,101,107]
Ctenophthalmus uncinatus (Wagner, 1898)	Poland [26,72], Slovakia [82,118]	Poland [72]	Slovakia [102]	
Rhadinopsylla integella Jordan et Rothschild, 1921	Austria [48], Czech (106], Slovakia [82,119]			
Rhadinopsylla isacantha (Rothschild, 1907)	Slovakia [120]			
Rhadinopsylla mesoides Smit, 1957	Slovakia [120]			
Rhadinopsylla pentacantha (Rothschild, 1897)	Slovakia [101,107], Hungary [117]			
**Siphonaptera, Hystrichopsyllidae**				
Hystrichopsylla orientalis Smit, 1956	Poland [26,60,105,113], Slovakia [82], Hungary [10-111,121]	Poland [60]	Poland [26,60,105], Slovakia [108]	Poland [60], Slovakia [115], Hungary [33,110,121]
Hystrichopsylla talpae (Curtis, 1826)	Poland [26,57,60,72,113], Austria [48,114], Czech [35,42,44,106], Slovakia [38,101]		Poland [60], Czech [42,44], Slovakia [101,107], Hungary [121]	
Doratopsylla dasycnema (Rothschild, 1897)	Germany [112], Poland [26,57,72,105,113], Austria [48,114], Czech [35,42,44,103,106], Slovakia	Poland [26,113], Austria [48], Czech [35,42,44,103], Slovakia [38,101,107,109,115],	Poland [26,73,105,113], Czech [42,44,103], Slovakia [101-102,107,108], Hungary [33,110]	Czech [44], Slovakia [101,107-108], Hungary [33,110-111]

	[22,38,82,101-102,107,115], Hungary [33,110-111,117]	Hungary [87]		
Palaeopsylla kohauti Dampf, 1911	Austria [48,114], Czech (106], Slovakia [101,122], Hungary [33,110]		Slovakia [101-102]	Hungary [110]
Palaeopsylla similis Dampf, 1910	Czech [35,42,44,106], Slovakia [33,101,107,109]		Poland [105], Slovakia [101,107]	Slovakia [82,102,107]
Palaeopsylla soricis (Dale, 1878)	Germany [112], Poland [26,57,71-73,104-105,113], Austria [48,114], Czech [35,42,44,103,106], Slovakia [22,33,38,82,101,102,107,115], Hungary [75-76, 78-79, 87]	Poland [26,104-105,113], Austria [48], Czech [35,42,44], Slovakia [101,107,109,115]	Poland [26,72-73,104-105,113], Czech [42,44,103], Slovakia [82,101-102,107-108], Hungary [33,40,110-111]	Poland [26,72,105], Austria [48], Czech [44,103], Slovakia [22,38,82,101-102,107], Hungary [110]
Palaeopsylla steini Jordan 1932	Czech [44], Slovakia [82]		Slovakia [82]	
**Anoplura**				
Hoplopleura acanthopus (Burmeister, 1839)	Poland [57,71-73], Slovakia [83,123-125]	Poland [126], Slovakia [124]	Poland [127], Slovakia [124]	Poland [72], Slovakia [124]
Hoplopleura affinis (Burmeister, 1839)	Slovakia [81,124-125]	Slovakia [124-125]		
Hoplopleura edentula Fahrenholz, 1916	Poland [57,71,127], Slovakia [82,124]	Slovakia [124], Hungary [28]	Poland [59], Slovakia [124]	Poland [59], Slovakia [124]
Polyplax reclinata (Nitzsch, 1864)	Slovakia [128], Hungary [28]	Slovakia [128], Hungary [28]	Hungary [28]	
Polyplax serrata (Burmeister, 1839)	Poland [71-73,127], Slovakia [124-125]	Poland [73], Slovakia [124]	Poland [59], Slovakia [124]	
